# Does post-bleaching fluoridation affect the further demineralization of bleached enamel? An in vitro study

**DOI:** 10.1186/1472-6831-14-113

**Published:** 2014-09-06

**Authors:** Hande Kemaloğlu, Hüseyin Tezel, Zeynep Ergücü

**Affiliations:** Department of Restorative Dentistry, Faculty of Dentistry, Ege University, Bornova, 35100 Izmir Turkey

**Keywords:** Bleaching, Demineralization, Sodium fluoride, Titanium tetrafluoride

## Abstract

**Background:**

Topical fluoride agents have been shown to be the most effective method in treating demineralized enamel after in-office bleaching treatments. Thus, this study aimed to examine the effects of two different post-bleaching fluoridation agents: 1.5% titanium tetrafluoride (TiF_4_) (9200 ppm) and 2.1% sodium fluoride (NaF) (9500 ppm), on the calcium loss of enamel after an acidic challenge.

**Methods:**

Ten maxillary premolars were sectioned into four pieces and then divided into the following four groups: Group 1: Control, kept in artificial saliva, no treatment; Group 2: 38% hydrogen peroxide (HP); Group 3: 38% HP followed by 1.5% TiF_4;_ Group 4: 38% HP followed by 2.1% NaF solution. The specimens were subjected to demineralization for 16 days, refreshing the solution every 4 days; that is, on the 4th, 8th, 12th, and 16th days. Calcium ion (Ca^2+^) concentration was determined by an atomic absorption spectrophotometer. Data were analyzed using Friedman and Wilcoxon tests (p = 0.05).

**Results:**

The loss of Ca^2+^ in each of the test groups was compared with that of the control group, depicting that there was a statistically significant difference among the groups after 4, 8, 12, and 16 days and in total (p < 0.05). The calcium released from the fluoride-applied groups was lower when compared with the 38% HP and control group. At the end of the 16th day, the total amount of calcium released from the TiF_4_-treated samples (9.12 mg/mL) was less than from the NaF-treated samples (13.67 mg/mL) (p < 0.05).

**Conclusions:**

Regarding the results of our *in vitro* study, the risk of further demineralization was significantly reduced with the use of TiF_4_ and NaF after bleaching with 38% HP. TiF_4_was found to be more effective in preventing Ca^2+^ release owing to acid attack when compared with NaF. In the case of an intra-oral acidic exposure, the use of topical 1.5% TiF_4_ and 2.1% NaF agents might be beneficial after bleaching with 38% HP.

## Background

Bleaching has been accepted as one method of treating discolored teeth. Recently, novel in-office bleaching products that use high concentrations of hydrogen peroxide (HP) have made in-office treatments easier. However, the effects of these products on enamel are still an open issue and need to be clarified. When vital teeth are bleached, as a result of the direct contact between the bleaching agent and the outer enamel surface, the enamel surface of the tooth crown can be affected by high levels of HP in bleaching agents, causing structural and morphological changes. There are many studies on the reduction in microhardness as well as the loss of calcium from bleached enamel [1–4]. Furthermore, some changes in bleached enamel were described as slightly erosive defects promoted by the bleaching agent [4–7].

The positive effect of highly concentrated fluoride products related to the inhibition of demineralization and erosion is well documented [8]. Different topical fluoride applications such as sodium fluoride (NaF), acidulated phosphate fluoride, and stannous fluoride are widely used in promoting enamel remineralization. However, unlike the commonly used agents, it has been suggested in the literature that titanium tetrafluoride (TiF_4_), may have a greater effect on enamel remineralization. Furthermore, the use of fluoride products after bleaching procedures has also been shown to be beneficial [2, 9, 10]. As topical fluoride is applied following bleaching, mineral loss is significantly reduced, microhardness is restored, and the resistance of enamel to demineralization is increased [4, 10, 11].

Fluoride has been confirmed to remineralize lesions by increasing resistance to acid attack by forming a calcium fluoride layer to inhibit demineralization [9]. In addition, the formation of a glaze layer has been shown when enamel surfaces were treated with low pH TiF_4_ [12, 13]. However, there are no data available in the literature on the preventive effect of these applications on further demineralization. Thus, the aim of this present study was to examine the effects of two different post-bleaching fluoridation agents (NaF and TiF_4_) on the Ca^2+^ loss from enamel after an acidic challenge. The null hypotheses tested were: (1) no difference will be observed in Ca^2+^ release between the fluoridated and non-fluoridated groups of bleached enamel surfaces, after being subjected to acidic attack; thus, these fluoride agents will not affect the susceptibility of bleached enamel to further demineralization and (2) no differences in Ca^2+^ release will be noted between NaF- and TiF_4_-treated enamel surfaces after being subjected to further demineralization following bleaching with 38% HP.

## Methods

This study was approved by the Ege University, Faculty of Medicine, Research Ethics Committee (19/10/2012) and written informed consent was received from participants.

### Sample preparation

Ten maxillary premolars extracted for orthodontic purposes at Ege University, Faculty of Dentistry were selected for this *in vitro* study. All participants gave written consent prior to the extraction process. The extracted teeth were rinsed in tap water, and cleaned of plaque and debris with a dental hand piece and brush. The buccal, palatal, and occlusal surfaces were checked under a stereomicroscope, and teeth with enamel defects or cracks were rejected. Ten selected teeth were stored in 0.9% NaCl and 0.1% thymol for 1 week at 4°C to eliminate the reproduction of microorganisms, and then rinsed with distilled water. Each tooth was sectioned bucco-palatally into two halves with a diamond disc. These halves were then sectioned longitudinally into two parts, so that four specimens were obtained from each tooth. These specimens were later randomly assigned to one of the four groups, on the condition that each part of every tooth would be in one of the four different groups (Table 1). Then, the teeth were covered with wax except for the enamel surface.

**Table 1:** **Test groups**

Groups	N	Bleaching agent	Fluoride application	pH	F application time
1	10	No agent	---------		---------
2	10	38% HP	--------		--------
3	10	38% HP	2.1% NaF	~1.2	1 minutes
4	10	38% HP	1.5% TiF_4_	~1.2	1 minutes

### Bleaching procedure

All specimens in three of the test groups were treated with a commercial in-office bleaching agent of 38% HP (Opalescence Xtra Boost; Ultradent, South Jordan, UT, USA) according to the manufacturer’s instructions. The untreated specimens in the fourth group were used as a control group and kept in artificial saliva (0.7 mmol/L CaCl_2_, 0.2 mmol/L MgCl_2_, 4.0 mmol/L KH_2_PO_4_, 30.0 mmol/L KCl, 20.0 mmol/L HEPES; pH 7.0) during the test period [14, 15].

A thick layer (~1 mm) of 38% HP (pH ≅ 7) was applied to the enamel surfaces of the specimens in the test groups (Table 1). To achieve optimum effectiveness, the bleaching gel was stirred/agitated every 5 min and refreshed every 15 min. The total time of application was 45 min per day. This procedure was repeated every other day for 3 days. After removing the whitening gel, the teeth were rinsed, dried, and kept in artificial saliva until the next procedure.

### Post-fluoridation process

Two out of the three test groups were treated with two different fluoride agents with approximately the same concentrations; 1.5% TiF_4_ (Aldrich Chem. Co, Milwaukee, WI, USA) (pH = 1.2, 9200 ppm) and 2.1% NaF (Merck, Switzerland) (pH = 1.2, 9500 ppm). They were applied for 60 s using a pipette while the third test group was left untreated and kept in artificial saliva during the test period after the bleaching process.

### Demineralization process

Immediately after the application of the bleaching and fluoride agents for the prescribed time, the specimens were rinsed with a water spray and dried with blasts of air. The enamel was then covered with standard “o”-shaped wax so as to expose a standard round window area (6.83 mm [2]) and acetic acid buffered with 0.34 M sodium acetate (pH = 4) was used as a demineralization buffer. A calcium monohydrate salt [Ca (H_2_PO_4_)_2_H_2_O)] was dissolved to obtain 10 mmol/L Ca^2+^ and 20 mmol/L PO_4_^3-^ in the solution [16].

Each specimen was treated with 50 mL of solution in polyethylene test tubes. The specimens were demineralized in four consecutive periods over 4 days. At the end of the 4th day, each specimen was taken out of the test tube and placed in a new tube, which contained fresh buffer solution. The previous solutions were kept in their tubes to be tested afterwards for their Ca^2+^ concentration using an atomic absorption spectrophotometer (AAS), as performed in previous studies [4, 16].

Calcium analysis was undertaken with the AAS using 0.1 mL of each demineralization solution, which was diluted with 4.9 mL of distilled water. To prevent the interaction of magnesium and phosphate ions, 50,000 mg/L of lanthanum chlorine (LaCl_2_) was added to each test tube to make up 10% LaCl_2_ in each buffer solution. The same procedure was applied to blank (buffer) and standard solutions of calcium. The amount of calcium released from tooth to buffer was calculated by measuring the difference in Ca^2+^. The calcium concentration in the samples was detected with an AAS (Varian Spectra-10 plus AA; Varian, Melbourne, Australia) (wavelength: 422.7 nm; slit 0.5 nm). The calcium released to the buffer after the 4th, 8th, 12th, and 16th days were compared using Friedman and Wilcoxon tests.

## Results

**Figure 1:**
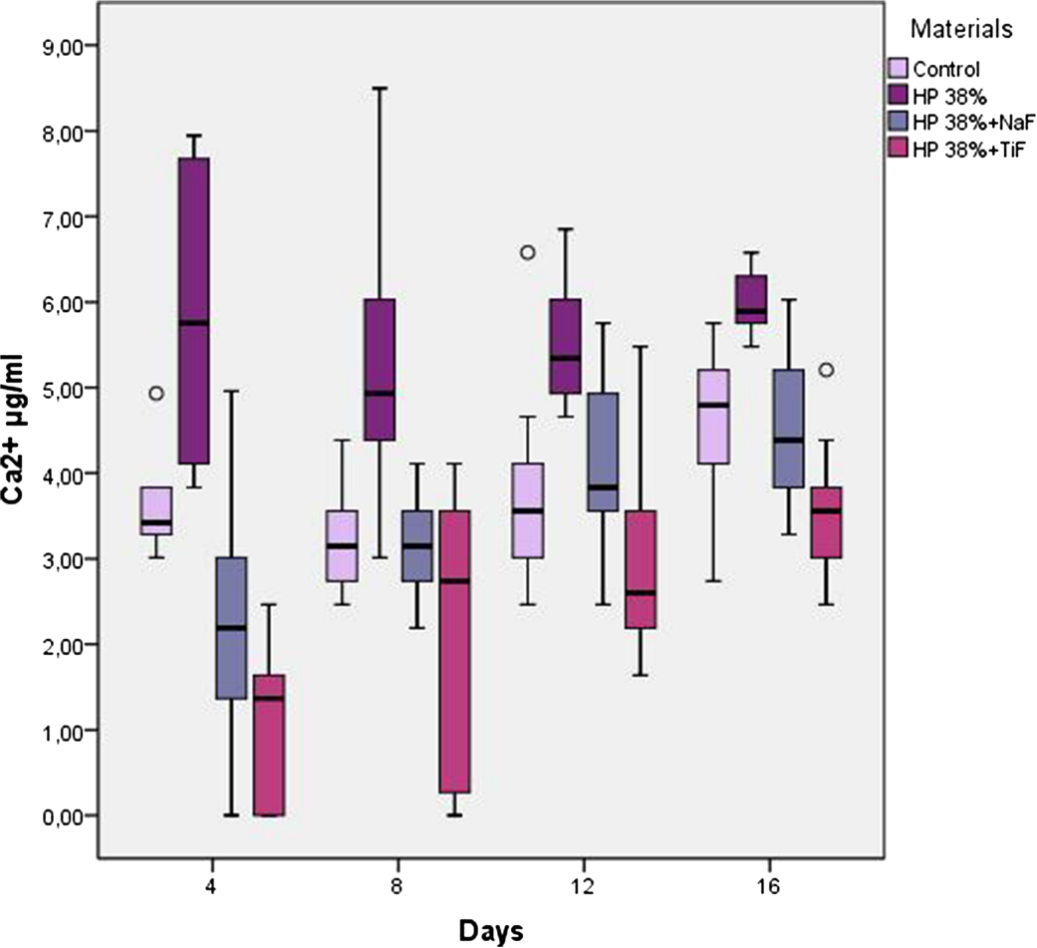
**The calcium ion (Ca** ^**2+**^ **) concentrations of the specimens measured at the end of the 4th, 8th, 12th, and 16th days (μg/mL).**

**Figure 2:**
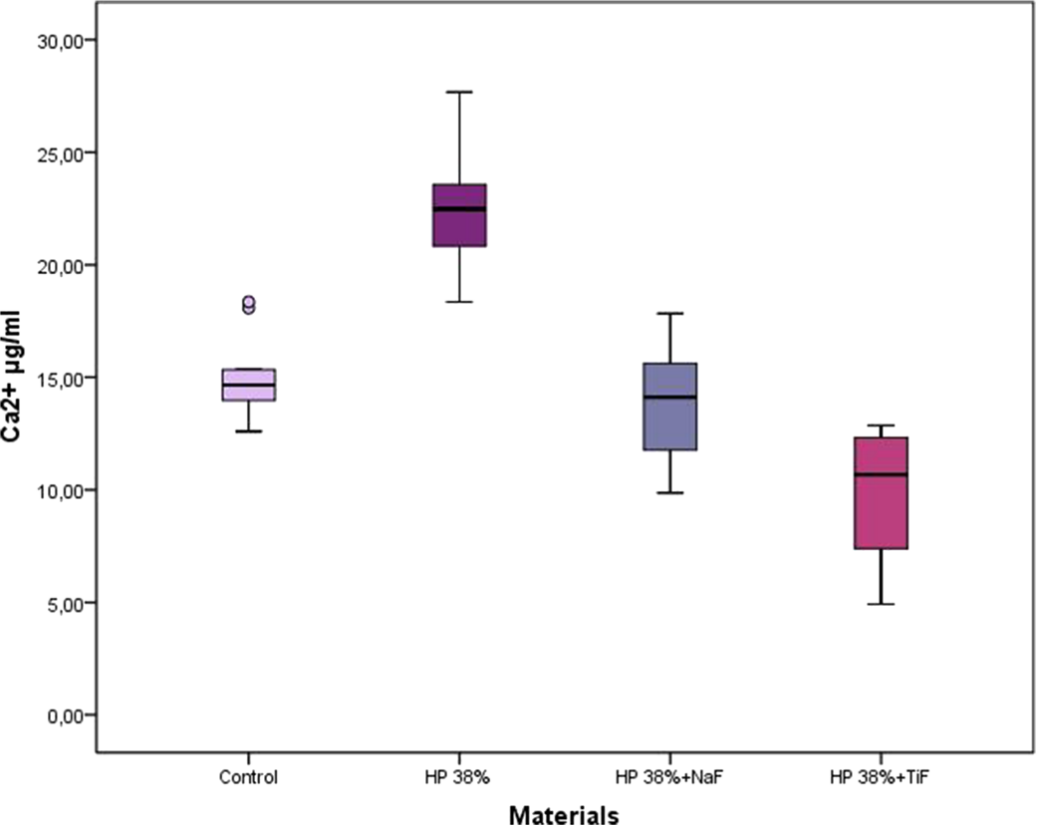
**The total calcium ion (Ca** ^**2+)**^ **concentrations of the specimens measured at the end of the 16th day (mg/ml).**

The Ca^2+^ concentrations of the samples were measured at the end of the 4th, 8th^,^ 12th, and 16th days (Table 2, Figure 1). The loss of Ca^2+^ in the control, 38% HP, 38% HP + NaF, and 38% HP + TiF_4_ groups were evaluated cumulatively every 4 days, and at the end of the 16th day, 15.07 ± 1.81 μg/mL, 22.44 ± 2.52 μg/mL, 13.67 ± 1.86 μg/mL, and 9.12 ± 2.40 μg/mL were obtained in total, respectively (Figure 2).

**Table 2:** **Calcium ion (Ca** ^**2+**^ **) release from the bleached specimens treated with 1.5% TiF**_**4**_ **and 2.1% NaF in mm** ^**2**^ **(mg/ml)**

	N		4th day	8th day	12th day	16th day	Total
Control group	10	Mean	3,59	3,2	3,72	4,55	15,07
		Std dev	0,54	0,59	1,23	0,89	1,81
		Min.	3,01	2,46	2,46	2,74	12,6
		Max.	4,93	4,38	6,58	5,75	18,36
38% HP	10	Mean	5,75	5,18	5,56	5,95	22,44
		Std dev	1,6	1,52	0,71	0,37	2,52
		Min.	3,83	3,01	4,66	5,48	18,36
		Max.	7,95	8,5	6,85	6,58	27,68
38% HP + NaF	10	Mean	2,05	3,15	3,94	4,52	13,67
		Std dev	0,83	0,58	1,01	0,83	1,86
		Min.	0,82	2,19	2,46	3,29	10,68
		Max.	3,01	4,11	5,75	6,03	15,61
38% HP + TiF4	10	Mean	1,15	1,94	2,66	3,37	9,12
		Std dev	0,92	1,54	0,63	0,48	2,4
		Min.	0	0	1,64	2,46	4,93
		Max.	2,46	4,11	3,56	3,83	11,5

The loss of Ca^2+^ in each of the test groups was compared with that of the control group using the Friedman test. A statistically significant difference was observed among the groups after 4, 8, 12 and 16 days and in total (p < 0.05). The Wilcoxon test was used to identify possible statistically significant differences between the groups.

After the demineralization process, there was significantly less Ca^2+^ released in the bleached/fluoride-treated groups (38% HP + NaF and 38% HP + TiF_4_) than in the bleached-only group (38% HP) and control group. When the NaF and TiF_4_-treated samples were compared, there were no significant differences between the amounts of Ca^2+^ released from the specimens after the 4th, 8th, and 16th days (p > 0.05). However, at the end of the test period, the total amount of Ca^2+^ in the buffer solution was significantly less for the TiF_4_-treated samples than for the NaF-treated samples (p < 0.05) (Tables 2 and 3). Thus, it might be suggested that TiF_4_-treated samples were more acid-resistant than NaF-treated samples.

**Table 3:** **Statistical differences between test groups**

Materials	4th day	8th day	12th day	16th day	Total
Control × 38% HP	0.007	0.017	0.022	0.005	0.005
Control × 38% HP + NaF	0.036	0.798	0.444	0.878	0.721
Control × 38% HP + TiF4	0.005	0.059	0.167	0.059	0.005
38% HP × 38% HP + NaF	0.009	0.005	0.022	0.009	0.005
38% HP × 38% HP + TiF4	0.005	0.013	0.007	0.005	0.005
38% HP + NaF × 38% HP + TiF4	0.074	0.241	0.047	0.059	0.017

Statistically significant differences between the groups (p<0.05).

No statistically significant differences (p>0.05).

## Discussion

High concentrations of HP that promote enamel surface alterations soften the superficial layer of the enamel surface, increase surface porosity, and release more Ca^2+^ than low concentrations of HP and carbamide peroxide (CP) [4, 15, 17]. Thus, 38% HP has been recruited for the present study to investigate the Ca^2+^ released from the bleached enamel surfaces after an acidic challenge. With this designated high concentration, it was aimed to observe the maximum Ca^2+^ release after a further demineralization process.

Alterations in the inorganic component of hydroxyapatite might be an indicator of the changes in Ca^2+^ levels of enamel. Rotstein *et al.* [1] demonstrated that most bleaching agents may cause various changes in the levels of calcium, phosphorus, and potassium in dental hard tissues; whereas Tezel *et al.* [4] demonstrated that 35% and 38% HP caused Ca^2+^ loss from the enamel surfaces. In the present study, Ca^2+^ released from the enamel of specimens treated with 38% HP was significantly higher than the untreated control group (p < 0.05). Based on the Ca^2+^ values, this result suggests that high concentrations of bleaching agents cause surface alterations after an acidic challenge.

The application of highly concentrated fluoride favors the formation of a CaF_2_-like layer [18]. This deposit is later dissolved, allowing fluoride to diffuse into the underlying enamel, saliva, or a plaque layer covering the tooth. It is assumed that some of the fluoride supports the remineralization of enamel. The results of a previous study confirmed that phosphates and proteins from saliva coated the calcium-fluoride layer on the enamel as a pH-controlling reservoir. This layer acted to decrease demineralization and promote remineralization [19].

Al-Qunaian *et al.* [20] investigated the effects of whitening agents on caries susceptibility of human enamel and reported that no significant differences in caries susceptibility were observed between the untreated control specimens and those specimens treated with 10% CP, 20% CP with fluoride, and 35% HP. There were no significant differences between the treated and controlled specimens for teeth treated with 10% CP or 35% HP. However, specimens treated with whitening gel containing 20% CP with fluoride had significantly reduced caries susceptibility when compared with their untreated controls. It was claimed that this effect could be related to fluoride incorporation in 20% CP gels containing fluoride, and the results were in agreement with laboratory studies that fluoride enhanced enamel remineralization.

In the present study, fluoride agents were applied to the bleached enamel and then subjected to further demineralization. When the test groups that were bleached with 38% HP were compared, the decrease in Ca^2+^ loss of the 1.5% TiF_4_-treated group was detected to be the lowest (Table 2). Regarding this result, it can be assumed that TiF_4_ may be effective in protecting the bleached enamel surface against acid attack. Interestingly, no Ca^2+^ release was detected from three specimens of TiF_4_ group during the first 4 days, and there was also no Ca^2+^ release from two specimens during the second 4-day interval (Table 2). We assume that this effect might be the result of the glaze formation after topical TiF_4_ application. It is known that formation of a glaze layer takes less than 10 s after the application of TiF_4_ [21]. The ability of TiF_4_ to strongly protect enamel against the action of acid is a synergistic effect of glaze formation and increased enamel fluoride content. The high fluoride content and great reduction in solubility found in TiF_4_-treated enamel suggests that a fluoride reaction with the enamel is involved [22]. In a previous study, Tezel *et al.* [16] reported that TiF_4_ was found to be more effective than Duraphat (NaF, 2.26% F) or Elmex (amine fluoride, 1.25% F) in preventing artificial enamel lesion formation. Attin *et al.* [23] reported that fluoridation was effective in increasing the resistance of enamel against demineralization by erosive substances. Similarly, the findings of this present study demonstrated that the resistance of bleached enamel against acid attack increased after 1.5% TiF_4_ treatment.

The comparison of the Ca^2+^ losses from the test groups that were bleached with 38% HP revealed that the decrease in Ca^2+^ losses of the 2.1% NaF-treated group was also lower, indicating that NaF could also protect enamel surfaces against acid attack. When the NaF-treated group was compared with the control group, it was seen that the amount of Ca^2+^ lost from the NaF group was significantly different during the first 4 days (p < 0.05) (Table 3). However, when the NaF-treated group was compared with the 38% HP group, the difference was statistically significant during the whole test period (16 days) (p < 0.05).

However, when the effect of NaF treatment against acid attack was compared with TiF_4_ treatment, it was observed that its influence was not as strong as TiF_4_ (Table 3, Figure 1). Tveit *et al.* [24] assumed that complexes were formed between TiF_4_ and hydroxyapatite, based on a strong binding of the titanium compound and the oxygen atom of the phosphate group. Mundorff *et al.* [22] suggested that TiF_4_ acted with enamel both chemically, by decreasing enamel solubility, and physically, owing to the formation of a protective glaze on the enamel surface. van Rijkom *et al.* [25] compared the erosion-inhibiting effect of topical fluoride treatment based on the deposition of CaF_2_-like material using 1% NaF and 4% TiF_4_. It was concluded that the reduction of Ca^2+^ loss was more stable for TiF_4_ than for the NaF group, and the reduction appeared to be smaller with longer acid exposure times.

Generally, fluoride uptake of demineralized enamel is higher when compared with sound enamel [26]. It is assumed that the applied fluoride can easily penetrate through the porous structure of demineralized enamel and that can create a higher number of possible retention sites [27, 28]. According to the results of one study [29], the bleached and fluoridated enamel was more resistant against erosive attacks than the bleached/unfluoridated and unbleached/unfluoridated enamel. In the present study, at the end of the test period, the total amount of calcium released from the bleached/fluoridated specimens was lower than the control group (unbleached/unfluoridated) and the difference was significant in TiF_4_-treated specimens (p < 0.05) (Table 3). This result is noteworthy in that bleached and fluoridated teeth may be more resistant to acid attack than sound teeth. These results would need to be investigated with further studies.

In the last decade, there has been a growing interest in demineralization and remineralization studies because of the demand for minimally invasive treatment techniques. When scanning through the literature, we encountered a number of different techniques applied in these types of studies. *In vitro* demineralization using acid buffers and *in vitro* demineralization/remineralization using a pH-cyling model are the most frequently used techniques that possess both advantages and disadvantages. It is important to choose the simplest and most practically appropriate model. Similar to the previous studies, we preferred using AAS for *in vitro* demineralization to observe the impact of fluoride agents on the Ca^2+^ loss following further demineralization. This method is a very sensitive but reliable method for calcium analysis, which avoids the interaction of other solutes [30, 31]. It can be used with confidence to quantify erosion of both enamel and dentine, and their chemical analyses of mineral release [32–34].

Based on the results of the present study, the null hypothesis that states there would be no difference between the amount of Ca^2+^ released from the enamel surfaces that were treated with NaF and TiF_4_ after an acidic challenge was rejected. It was also shown that topical fluoride application decreased the amount of Ca^2+^ released from the 38% HP-treated enamel surfaces after further demineralization. TiF had a significantly more pronounced effect than NaF in protecting enamel surfaces against acidic attack after bleaching with 38% HP.

## Conclusions

It may be concluded that post-bleaching fluoride application may be beneficial in reducing the risk of demineralization caused by acid attack after bleaching processes and to remineralize the bleached enamel surfaces. In addition, TiF_4_-treated samples released less Ca^2+^ than NaF-treated samples, which indicates that TiF_4_ may be more effective than NaF in preventing damage from acid attack.
